# Prevalence and Knowledge Assessment of HIV and Non-Communicable Disease Risk Factors among Formal Sector Employees in Namibia

**DOI:** 10.1371/journal.pone.0131737

**Published:** 2015-07-13

**Authors:** Leonor Guariguata, Ingrid de Beer, Rina Hough, Pancho Mulongeni, Frank G. Feeley, Tobias F. Rinke de Wit

**Affiliations:** 1 PharmAccess Foundation, Amsterdam, the Netherlands; 2 PharmAccess Foundation, Windhoek, Namibia; 3 Boston University School of Public Health, Boston, Massachusetts, United States of America; 4 Amsterdam Institute for Global Health and Development (AIGHD), Academic Medical Center, University of Amsterdam, Amsterdam, the Netherlands; Tulane University School of Public Health, UNITED STATES

## Abstract

**Introduction:**

The burden of non-communicable diseases (NCDs) is growing in sub-Saharan Africa combined with an already high prevalence of infectious disease, like HIV. Engaging the formal employment sector may present a viable strategy for addressing both HIV and NCDs in people of working age. This study assesses the presence of three of the most significant threats to health in Namibia among employees in the formal sector: elevated blood pressure, elevated blood glucose, and HIV and assesses the knowledge and self-perceived risk of employees for these conditions.

**Methods:**

A health and wellness screening survey of employees working in 13 industries in the formal sector of Namibia was conducted including 11,192 participants in the Bophelo! Project in Namibia, from January 2009 to October 2010. The survey combined a medical screening for HIV, blood glucose and blood pressure with an employee-completed survey on knowledge and risk behaviors for those conditions. We estimated the prevalence of the three conditions and compared to self-reported employee knowledge and risk behaviors and possible determinants.

**Results:**

25.8% of participants had elevated blood pressure, 8.3% of participants had an elevated random blood glucose measurement, and 8.9% of participants tested positive for HIV. Most participants were not smokers (80%), reported not drinking alcohol regularly (81.2%), and had regular condom use (66%). Most participants could not correctly identify risk factors for hypertension (57.2%), diabetes (57.3%), or high-risk behaviors for HIV infection (59.5%). In multivariate analysis, having insurance (OR:1.15, 95%CI: 1.03 – 1.28) and a managerial position (OR: 1.29, 95%CI: 1.13 – 1.47) were associated with better odds of knowledge of diabetes.

**Conclusion:**

The prevalence of elevated blood pressure, elevated blood glucose, and HIV among employees of the Namibian formal sector is high, while risk awareness is low. Attention must be paid to improving the knowledge of health-related risk factors as well as providing care to those with chronic conditions in the formal sector through programs such as workplace wellness.

## Background

The burden of non-communicable disease (NCDs) is growing in sub-Saharan Africa[1,2] and Namibia in particular is heavily affected[1]. The World Health Organization indicates that Namibia has the 2^nd^ highest rate of deaths attributable to cardiovascular disease and diabetes in the African region and is in the top 20 globally. The country also has the 4^th^ highest mortality attributable to NCDs in the African region, a prevalence of 9.2% of raised blood glucose, and the 8^th^ highest prevalence of high blood pressure in the world (49.1%)[1]. This growing problem is coupled with the already high burden of infectious diseases like HIV and tuberculosis. The prevalence of HIV in Namibia among people aged 15–49 is estimated at 13.1% or 160,000 people aged 15 and over[3].

For employers, maintaining the health of employees translates to reduced costs[4–6]. Studies have shown that poor health among employees leads to decreases in productivity and increased absenteeism[7–10]. Health promotion programs and the adoption of employer-paid or contributed health insurance can lead to decreased absenteeism and reduced costs for employers[4,11–14].

Three conditions which have emerged as serious contributors to the health burden of Namibia (hypertension, diabetes, and HIV) have all been shown to be preventable through education on risk factors, lifestyle changes and behavior modification programs, and regular screening and education[15–21]. As a contribution to this, the formal sector employment in Namibia represents an opportunity to provide education and screening through company wellness programs, which can in turn reduce costs and improve the health of employees and complement public sector efforts in these areas.

The purpose of this analysis was to conduct a secondary analysis of a large dataset derived from a workplace wellness survey to estimate the prevalence of three conditions in the study population: elevated blood pressure, elevated blood glucose, and HIV. In addition, this study puts the biomedical findings in the context of the knowledge and self-perceived risk of employees for these conditions and their related risk behaviors and determinants in order to identify areas for improvement in education and screening.

## Methods

The study is based on a secondary analysis of data obtained from a health and wellness screening survey conducted of 11,192 participants in the Bophelo! Project in Namibia, from January 2009 to October 2010. The survey is the largest ever performed in the formal sector in Namibia and reached employees in 13 industries, including self-reported data as well as a biomedical assessment. Information was collected per sector of each company as well as whether the company had an HIV prevention and education workplace program in place including services such as regular information sessions and free voluntary screening. Participation in the screening and survey was entirely voluntary and strictly confidential. To protect confidentiality, no identifiers were stored with the information gathered and participants gave written informed consent before inclusion.

The survey was performed by two mobile clinics from the Bophelo! Project, a partnership between PharmAccess, the Namibian Business Coalition on AIDS and the Namibia Institute of Pathology. As part of the project, wellness surveys and health screening services were offered to companies along with sensitization and information sessions provided before employee screenings. The mobile clinic visited a company site for as long as required to see all participating employees (up to 32 people per mobile clinic per day) and each screening visit took approximately a total of 45–60 minutes consisting of pre-test counseling (30–45 minutes), testing, and post-test counseling sessions. All services were provided by Ministry of Health and Social Services (MOHSS)-trained testers and counselors. While screening results were being obtained, the participant received health education and confidential counseling. Participants who tested positive for any condition or had screening results outside of a normal range were encouraged to seek follow-up consultation and were issued a referral letter to local medical services. Only de-identified information was collected from the screenings without any possibility of linking back to the participant. The study was approved by the ethical board of the MOHSS of Namibia.

Participants who provided informed consent were asked to complete a questionnaire with demographic (age, sex, marital status, smoking), job status information (contract type, type of position), and knowledge, self-perceived risk and self-reported presence of the conditions: HIV/AIDS, diabetes, hypertension and cardiovascular disease. Knowledge of conditions was assessed by asking participants to respond to true-false statements about related symptoms and risk behaviors. For hypertension, participants were asked whether the following activities could lower their risk of hypertension: maintaining a healthy body weight, increasing physical activity, avoiding salt in the diet, stopping smoking, avoiding excessive alcohol intake, and whether or not it was possible for an individual to lower their own risk of hypertension. Similarly, participants were asked about diabetes-related risk behaviors including: maintaining a healthy weight, increasing physical activity, and lowering one’s own risk for diabetes. Questions for HIV/AIDS risk were: whether a mother could transmit HIV to her child, whether mother-to-child transmission could be prevented, if condom use can prevent HIV infection, identifying true and false statements regarding transmission routes for HIV including food sharing, shaking hands, kissing, and sexual behavior. In addition, participants were asked whether a healthy looking person could have HIV, whether a person with tuberculosis always has HIV, whether traditional healers can cure HIV/AIDS, and whether having sex with a virgin can cure AIDS.

For the purposes of analysis, “good” knowledge of the conditions was considered a correct answer on each of the respective questions. Knowledge was therefore calculated as a binomial variable (All correct answers/Not all correct answer) for use in univariate and multivariate analysis.

In addition, participants who agreed received a medical screening for HIV status, random blood glucose, systolic and diastolic blood pressure and anthropometric markers. A finger prick was conducted on all participants to collect a blood sample for HIV and blood glucose. The following assays and methods were used for collecting medical screening information.

### HIV Testing

The Determine HIV 1/2 Assay by Inverness rapid test strip and Trinity Biotech Uni-Gold HIV 1/2 test kit were used in conjunction for HIV testing, according to Namibian VCT regulations. If the result was discordant between the two tests, a third (tie-breaker) test was performed using the Inverness Clearview Complete HIV 1/2 test kit.

### Blood glucose test

Blood glucose was assessed through a non-fasting capillary blood draw. Values outside a normal range (3.0 mmol/L– 6.6 mmol/L) were repeated by a second draw and the second result taken for analysis. The Accutrend Plus GCT meters were used to conduct the test (Roche Molecular Diagnostics, USA). The meters were operated at 18 to 32 degrees Celsius. Diabetes was considered likely if the result was ≥11 mmol/L in accordance with the American Diabetes Association guidelines, and an elevated value was considered a result ≥ 6.6 mmol/L. Participants with values ≥ 11 mmol/L were referred for follow-up to a medical facility.

### Blood pressure determination

Systolic and diastolic blood pressure was measured using a sphygmomanometer in millimetres of mercury (mmHg) by using the MG150f Digital Blood Pressure monitor (Rossmax International Ltd, Taiwan). Blood pressure was measured on the upper arm, in a sitting position. The measurement was taken three times on each participant and the average of the three readings was used. A blood pressure reading above 140/90 mmHg was considered elevated; above 153/103 mmHg was considered high and referral was made to a medical facility.

### Statistical Analysis

Data were collected in DOS and stored using SPSS. All statistical analyses were conducted using R Project for Statistical Computing version 2.10.0 (www.r-project.org). The chi-squared test for significance was used for comparisons in contingency tables with p ≤ 0.05 considered statistically significant.

Participants were allowed to refuse any or all of these screenings. Not all subjects took every test because either they refused to have it, or the employer opted not to provide the particular screening to that company. In this case, the subjects were excluded from analysis for a particular screening and only respondents included.

Multivariate analysis was carried out using logistic regression analysis. Where necessary, variables with several categories were modified to binary variables for ease of interpretation in multivariate analyses and where the additional information provided by the categories was not significant.

## Results

### Demographics, self-reported health, and health-related behaviors

The majority of survey participants were below the age of forty, male, and had not completed a secondary school level education (Table 1). In addition, most were working in manual labor and not in administration, management, or supervisory positions. The majority of respondents worked in food manufacturing and fishing.

**Table 1:** Demographic and screening results for hypertension, high blood glucose and HIV of formal sector employees in Namibia.

Demographics				
		N	%	95%CI
Age				
	20–29	3,150	28.1	(27.3–29.0)
	30–39	3,524	31.5	(30.6–32.4)
	40–49	2,363	21.1	(20.4–21.9)
	50–59	1,154	10.3	(9.8–10.9)
	≥60	226	2	(1.8–2.3)
Sex				
	Male	7,356	65.7	(64.8–66.6)
	Female	3,834	34.3	(33.4–35.1)
Education				
	Below Secondary	5,078	45.4	(44.4–46.3)
	Secondary or Higher	5,073	45.3	(44.4–46.2)
Job Type				
	Administration	976	8.7	(8.2–9.3)
	Management	552	4.9	(4.5–5.3)
	Supervisor	648	5.8	(5.4–6.2)
	Labour	8,732	78	(77.2–78.8)
Sector				
	Administration and Business	408	3.6	(3.3–4.0)
	Hotel and Restaurants	331	3.0	(2.7–3.3)
	Food Manufacturing	2,504	22.4	(21.6–23.1)
	Retail and Trade	1,145	10.2	(9.7–10.8)
	Tourism	525	4.7	(4.3–5.1)
	Wholesale	245	2.2	(1.9–2.5)
	Agriculture	1,112	9.9	(9.4–10.5)
	Utilities	1,264	11.3	(10.7–11.9)
	Infrastructure	691	6.2	(5.7–6.6)
	Fishing	1,969	17.6	(16.9–18.3)
	Manufacturing	63	0.6	(0.4–0.7)
	Mining	318	2.8	(2.5–3.2)
	Storage and Transport	617	5.5	(5.1–5.9)
Screening Results				
		N	%	95%CI
Blood pressure				
	≥140/90 mmHg (Elevated/High)	2,888	25.8	(25.0–26.6)
	<140/90 mmHg (Normal)	8,223	73.5	(72.7–74.3)
	Declined or Not Tested	81	0.7	(0.6–0.9)
Blood glucose				
	≥ 6.6 mmol/L (Elevated)	933	8.3	(7.8–8.8)
	<6.6 mmol/L (Normal)	9,583	85.6	(85.0–86.3)
	Declined or Not Tested	676	6.0	(5.6–6.5)
HIV				
	Positive	993	8.9	(8.4–9.4)
	Negative	8,848	79.1	(78.3–79.8)
	Declined or Not Tested	1,351	12.1	(11.5–12.7)

For self-reported health status, almost 80% reported being in “good” or “excellent” health (n = 8,730). Ten per cent of participants reported having hypertension. Furthermore, 25.8% found to have an elevated blood pressure on screening. Fifty-nine per cent of respondents who reported being diagnosed with hypertension were female. Less than 1.5% of participants reported having been diagnosed with diabetes.

With regard to risk behaviors, the majority of participants reported eating fruits daily, 20% reported being smokers and 19.8% reported drinking alcohol once per week. The majority of participants reported they did not have sex outside their regular relationships (76.6%) and the majority also reported using a condom always if having extra-marital sex (66.0%). A total of 8.9% of respondents tested positive for HIV upon screening.

### Self-perceived risk

**Fig 1:**
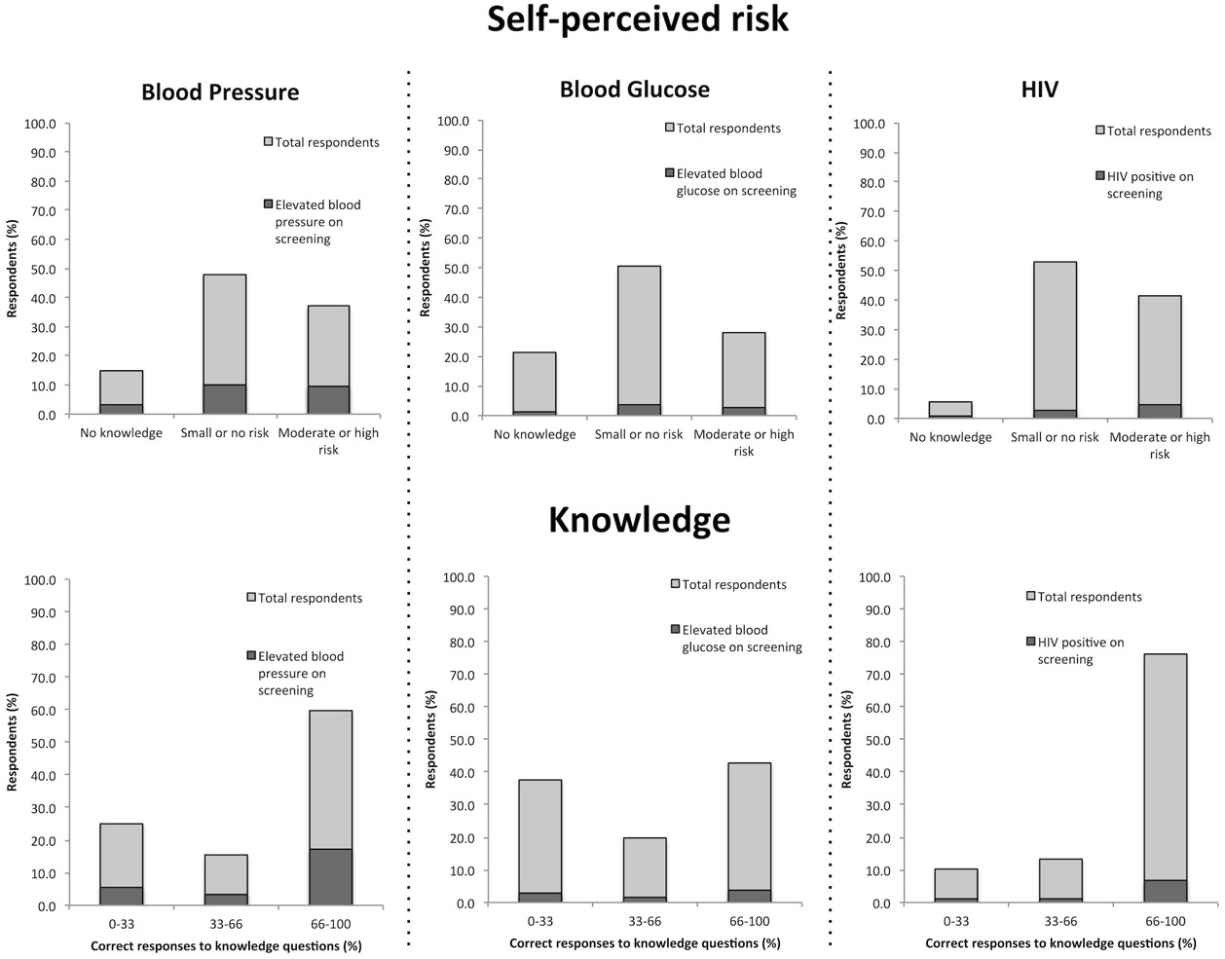
Self-perceived risk and knowledge of hypertension, diabetes, and HIV among total respondents and those with abnormal screening results in Namibian formal sector employees. Light grey bars represent responses among the total population, while the dark grey bars indicate what proportion of those respondents had abnormally high values on screening for blood pressure and blood glucose, or positive HIV screening.

For all three conditions, excluding non-respondents and those who reported having the condition, the majority of respondents reported no knowledge of or felt they had no risk or a small risk of developing the condition (Fig 1). With regard to self-perceived risk of each condition, almost half of respondents considered they had a low to no risk of hypertension (48.1%), or did not know what the condition was (14.9%). Similarly, 44.5% of respondents thought they had low or no risk of developing diabetes, and 14.0% reported they did not know anything about diabetes. Conversely, only 5.7% of reported no knowledge of HIV/AIDS and 41.5% of respondents thought they had an at least moderate risk of contracting HIV.

When factoring in screening results, 21% of those who reported they had no knowledge of hypertension were found to have elevated blood pressure. Similarly, 21% of those reporting no knowledge of HIV tested positive upon screening. Nine per cent of those reporting no knowledge of diabetes were found to have elevated blood glucose on screening.

### Knowledge assessment

Participants were asked a series of questions relating to each of the three conditions. For questions related to hypertension, 42.8% of participants correctly responded to all questions on risk factors and associated behaviors for hypertension (Fig 1). A significantly larger proportion of those previously diagnosed with hypertension were able to correctly identify the risk factors compared to the rest of respondents (55.9% vs. 41.3% respectively, χ^2^ = 86.2, p<0.05). Similarly, 42.7% of participants were able to correctly answer all questions relating to the risk of diabetes and those previously diagnosed were significantly more likely to answer diabetes-related questions correctly than the rest of respondents (65.6% vs.42.5%, χ^2^ = 37.4, p<0.05) (Fig 1). 3.2% of all respondents found on screening to have elevated blood pressure had no knowledge of the condition. Similarly, 1.3% of all respondents with elevated blood glucose on screening had no knowledge of diabetes, and 0.6% of those with positive HIV screening reported no knowledge of HIV.

Less than half of participants were able to correctly answer all questions correctly related to risk factors and behaviors for HIV/AIDS (40.5%), although there were more questions (n = 11) assessed than for diabetes (n = 3) or hypertension (n = 6). In addition, 95% of respondents were able to answer at least two-thirds of HIV-related knowledge questions correctly (Fig 1), compared to 64% for hypertension and 48% for diabetes. Those who screened positive for HIV were slightly better at answering behavior questions correctly (42.5%) compared to those who screened negative (39.9%; χ^2^ = 6.82, p = 0.03).

### Univariate and multivariate knowledge analysis

On univariate analysis and including only industry as a covariate to account for any clustering, increasing age and having insurance were significantly associated with good knowledge of both hypertension and diabetes (Table 2). Having an administrative or managerial position was associated with better odds of good knowledge for all three conditions (Table 2). In addition, being female was associated with significantly better knowledge of HIV compared to men (Table 2).

**Table 2:** Factors associated with knowledge* of hypertension, diabetes and HIV among formal sector employees in Namibia.

	Crude Model: OR (95% CI)			Adjusted Model: OR (95% CI)		
	Hypertension	Diabetes	HIV	Hypertension	Diabetes	HIV
Variables						
Age (continuous)	1.02 (1.02, 1.02)	1.01 (1.01, 1.02)	1.00 (0.99, 1.00)	1.02 (1.02, 1.03)	1.02 (1.01, 1.02)	1.00 (0.99, 1.00)
Sex (Female vs. Male)	1.08 (0.99, 1.18)	1.04 (0.96, 1.14)	1.29 (1.19, 1.41)	1.09 (0.99, 1.21)	1.02 (0.92, 1.12)	1.26 (1.14, 1.39)
Education (≥ Secondary vs. Below Secondary)	0.91 (0.84, 0.99)	0.99 (0.91, 1.07)	1.02 (0.94, 1.11)	0.90 (0.82, 1.00)	0.93 (0.84, 1.02)	0.92 (0.83, 1.01)
Insrance (Yes vs. No)	1.15 (1.05, 1.26)	1.28 (1.17, 1.40)	1.02 (0.93, 1.12)	1.06 (0.95, 1.18)	1.15 (1.03, 1.28)	0.97 (0.87, 1.08)
Job type (Adminstrative/Managerial vs. Manual labour)	1.11 (1.00, 1.23)	1.37 (1.24, 1.52)	1.12 (1.01, 1.24)	1.03 (0.90, 1.18)	1.29 (1.13, 1.47)	1.11 (0.97, 1.27)

*Knowledge is defined as correctly answering all questions related to each of the three conditions on the given survey. The crude model included industry as a covariate. The adjusted model further included all the covariates listed (age, sex, education, insurance, job type).

In multivariate analysis controlled additionally for age, sex, education, insurance, and job type (Table 2). After controlling for covariables, increasing age was associated with good knowledge of hypertension and diabetes; being female was associated with better knowledge of HIV; and having insurance or having administrative position were associated with diabetes knowledge (Table 2). None of the independent variables was strongly associated with better knowledge of a particular disease. No other significant associations or trends were for any of the other independent variables described for any of the three conditions.

## Discussion

The Bophelo! Project survey presented here covered the majority of key industries in Namibia and the sample size represents approximately 3% of the total Namibian formal sector workforce[22]. The results presented in this paper demonstrate overall low knowledge and self-perceived risk for three major conditions among a large Namibian population working in the formal sector. Those who were previously diagnosed showed overall better knowledge of their conditions than total respondents, suggesting that some education by their care providers is reaching them. Of the three conditions, respondents demonstrated better overall knowledge of HIV, which may reflect efforts by the MOHSS in population wide HIV-related education. Knowledge on blood pressure and blood glucose was overall low. An assessment of African migrants in Glasgow showed a similar pattern with low knowledge of chronic diseases and infectious disease perceived as the greatest threat[23]. Low knowledge levels could also be partly explained by the fact that the overall disease burden of NCDs in Namibia is lower and has a shorter history than that for HIV[24,25], but there is an expectation of a rapid rise in CVD and diabetes for low- and middle-income countries in the next generation[26].

Self-perceived risk has also been shown to affect health-seeking behavior and behavior change. A study of perceptions of risk of HIV/AIDS and sexual behavior in Kenya found a strong positive association between self-perceived risk and risky sexual behavior[27]. Where there is discordance between self-perceived risk and actual risk, the necessary treatment may be delayed or risk behaviors amplified; a relationship that has been seen in studies of HIV[19,28,29] as well as diabetes[30,31] and hypertension[32] in African populations. In this study, majority of participants were not able to accurately assess their own risk of any of the three conditions presented regardless of screening result which may influence not only health-seeking behavior, but also the self-monitoring and management necessary to prevent complications related to any of the three conditions.

From multivariate analysis, it is difficult to determine the independent factors related to gaps in knowledge from demographic and work-related information. None of the independent variables had a strong relationship with knowledge of a particular condition. Studies have shown some benefit to workplace wellness education programs in improvements on cardiac risk factors[6], obesity[14], cardiovascular disease and diabetes[33], and physical activity[34]. In general, Rula and Hobgood reported that “workplace health promotion programs founded on objective health metrics can motivate employee health-risk reduction”[35]. However, targeted education may be more effective. Abubakari et al. found that diabetes knowledge and illness perception varied by ethnicity, socioeconomic status, and other demographic factors[30], and similar results have been seen with self-reported health status and NCD risk factors in the US[36]. Further research is needed to determine target groups and those who would be particularly vulnerable to gaps in knowledge among formal sector employees.

This study demonstrates the gaps in knowledge related to three conditions: diabetes, hypertension, and HIV, which have been shown to have significant impacts on employees and employers[7, 12, 25–28]. Directed workplace programs may provide the best opportunity for closing such knowledge gaps. Work environments may provide a venue for screening and education regarding healthy lifestyles and behaviors. Research has shown that introducing wellness programs in the workplace can have benefits not only for the individual and their ability to maintain a healthy lifestyle and reduce risks for certain disease, but also as a way for employers to reduce costs related to absenteeism and lost productivity from illness[6,13,37]. Evidence for all three conditions shows that early intervention after diagnosis can significantly help prevent complications and progression[15,17,20], especially in an asymptomatic phase. These data could also be used to encourage employers to expand existing workplace programs focused on one disease to include education on other conditions and risk behaviors.

### Limitations

Because this is a cross-sectional survey, it is impossible to determine causation and only associations between independent variables and the outcomes (knowledge) can be ascertained. In addition, the survey was meant as an awareness and management information exercise for companies and was not powered to detect particular associations. This increases the potential for type II statistical errors or the probability of finding a significant result when one does not actually exist. In addition, the screening procedures are not the best standard in studies for prevalence of diabetes, hypertension, or HIV. A random blood glucose test, in particular, is the least sensitive measure and may be subject to large variations depending on when the subject last ate, their level of physical activity, and other factors. As a result, the screening results for high blood glucose should be interpreted with caution and are only an indication of possible elevated blood glucose rather than providing a basis for studying prevalence. Similarly, diagnostic standards for hypertension require that blood pressure be measured on two separate occasions to confirm a diagnosis. The scope of the wellness survey, as well as the resources available for the study precluded more rigorous screening measures. These do, however, represent the first indication of measures of risk factors for NCDs in a large Namibian population as far as the authors are aware.

Because participation was voluntary, there may be a selection bias, which would make results not applicable to the general population. No particular adjustments were made for missing data as they were not found to be systematically distributed throughout the sample. None of the variables had a greater than 5% proportion of missing data. While this survey collected information on those who refused to learn their results, it did not provide information on those who refused to have the test done at all. Given the sample size of this largest survey amongst Namibian formal sector employees, the results could be interpreted as the best available representative health data in the Namibian formal sector to date.

## Conclusions

Despite increasing awareness of chronic conditions in sub-Saharan Africa, knowledge among formal sector workers in Namibia is poor. Opportunities exist for improving the knowledge and risk perception of NCDs and HIV among formal sector employees in this country. However, more research is needed to ascertain prevalence of these conditions and to determine the most effective strategies for management and prevention.
